# What Do the Managers Think of Us? The Older-Worker-Perspective of Managers’ Attitudes

**DOI:** 10.3390/ijerph18084163

**Published:** 2021-04-14

**Authors:** Annette Meng, Emil Sundstrup, Lars L. Andersen

**Affiliations:** National Research Centre for the Working Environment, 2100 Copenhagen, Denmark; ESU@nfa.dk (E.S.); LLA@nfa.dk (L.L.A.)

**Keywords:** ageism, social psychology, aging at work, retaining older workers, the SeniorWorkingLife study, age stereotypes

## Abstract

Background: Due to demographic changes, the need to prolong working life has become increasingly salient. Paradoxically, stereotyping and discrimination against workers based on their age can limit possibilities for a long working life. A large body of research has investigated attitudes towards older workers; however, less is known about differences across occupational groups as well gender differences. Aim: To compare perceptions of the managers’ perceptions of older workers between employees from mainly seated work and mainly physical work as well as men and women. Method: Data from 11,444 workers aged 50+ from the baseline questionnaire survey in the SeniorWorkingLife study were analyzed. Results: Across all groups, a larger proportion of the respondents indicated that their managers had more positive than negative perceptions of older workers. Respondents from the International Standard Classification of Occupations (ISCO) group 5–9 (mainly physical work) and women were less likely to point at both positive and negative perceptions than the ISCO group 14 (mainly seated work) and men, respectively. Conclusions: The results indicate that there are differences between those with mainly physical and mainly seated work as well as gender differences. More research is warranted to explore the nature of these differences and, in particular, attitudes towards older female and male workers, respectively.

## 1. Introduction

Due to the demographic changes, the proportion of older workers in the labor market is increasing, and thereby the proportion of the workforce entering retirement age is likewise increasing. To prevent a shortage of qualified labor and to deal with the economic strain associated with these demographic changes, the need to prolong work life has become increasingly salient. A measure to prolong working life in many western countries includes increasing the retirement age [1]. Nevertheless, this measure cannot stand alone. It is necessary to avoid that older workers are pushed out of the labor market before retirement age, and to motivate them to continue working until retirement age or perhaps even longer. Older workers are commonly defined as those aged 50 years or older [2,3,4], although this definition may be fluid and change with the context and over time. Research has found that attitudes and stereotypes about older workers influence managers hiring intentions and decisions [5,6,7], and thereby their opportunities to re-enter the labor market if they are unemployed, which again increases the risk of these older workers retiring prematurely. In addition, negative stereotypes towards older workers are associated with lower level of work engagement [8], and ageism is associated with intention to retire early [8,9,10,11]. Negative attitudes towards older workers thus represents an obstacle to prolonging working life.

A large body of research has investigated stereotypes held by managers about older workers, and both negative and positive stereotypes have been identified [12,13,14,15,16]. The negative stereotypes include that older workers are less motivated, less productive, less adaptable and flexible, more resistant to change, harder to train and have a lower ability to learn, have shorter job tenure, and are more costly [17]. The positive stereotypes include that older workers are more stable, dependable, honest, trustworthy, loyal, committed, are less likely to miss work or turnover quickly [17]. Several studies find that employers or managers to a large extent hold positive attitudes towards older workers [15,18,19,20]. Nevertheless, Van Dalen, Henkens [14] distinguishes between “soft skills”, comprising reliability, loyalty, social skills, and management skills, and “hard skills” comprising stress resistance, creativity, flexibility, stamina, new tech skills, and the willingness to train. They found that older workers are often rated higher on soft skills than hard skills, putting them at a disadvantage in recruitment situations because the hard skills are valued higher.

In addition, findings indicate that older managers [13,21,22,23] and managers that are in more frequent contact with older workers [13] hold more positive attitudes towards older workers. A recent study found that concrete experiences with older workers influenced the managers′ perceptions of older workers [24], indicating that it is not enough merely to have frequent contact with older workers to develop more positive attitudes towards them; the concrete experiences have to be positive.

Furthermore, there are indications in the literature that both the extent and nature of the attitudes towards older workers among managers vary across occupational groups [17,19,23,24,25,26], and that that managers’ attitudes towards older workers may be less negative in white-collar jobs [21,22]. Furthermore, qualitative results illustrate that the nature of the negative attitudes may also differ between physically demanding jobs and white-collar jobs. The workability was considered to decrease in physically demanding jobs, while in white-collar jobs, age was expressed as an obstacle to the mental attitudes of the older workers—“they mentally retire after the age of 50” [27]. All in all, these findings indicate that the attitudes towards older workers differ between occupational groups. Nevertheless, the majority of these studies are qualitative and thus based on very small sample sizes, or differences between occupational groups are not compared systematically. Therefore, research based on large samples exploring differences between occupational groups in managers’ attitudes towards older workers has been called for [26]. Greater knowledge about differences between occupational groups in which attitudes or stereotypes are widespread will help inform targeted interventions to eliminate negative attitudes towards older workers.

In addition, the attitudes and stereotypes may depend on whether the target is older female or male workers. Research shows that there are gender stereotypes about workers [28,29], and there is no reason to believe that these disappear as the workers are getting older, but they may change and may change differently for men and women. Findings indicate that attitudes towards older workers are gender-specific both in regards to the gender of the employer and the gender of the older workers [7]. They found that the gender of the employer affected the rating of older women’s interest in competence development, but not the rating of older men’s interest in competence development. Nevertheless, the gender of the older workers has largely been ignored in research on stereotypes or attitudes towards older workers. Once again, a more detailed knowledge of attitudes towards older workers and differences between subgroups of older workers, such as occupational group and gender, will help inform targeted interventions to eliminate negative attitudes and stereotypes, and potentially contribute to the prevention of early retirement.

In the SeniorWorkingLife study, data from a large representative sample of employees aged 50+ were collected. Amongst others, they were asked to indicate whether they believed the managers at their workplace had a list of both positive and negative perceptions of older workers, thus providing an indirect measure of the managers’ attitudes towards older workers.

Based on these data, the study aimed to answer the following two research questions:

(1) Are there differences between mainly seated work (International Standard Classification of Occupations (ISCO group 1–4) and mainly physical work (ISCO group 5–9) in the proportion of respondents perceiving managers to have negative and positive perceptions of older workers, respectively?
(2) Are there differences between women and men in the proportion of respondents perceiving managers to have negative and positive perceptions of older workers, respectively?

## 2. Methods

### 2.1. Design and Sample

In this article, we report findings based on the baseline questionnaire survey from 2018 of the SeniorWorkingLife study, and thus apply a cross-sectional study design. The study is registered as a cohort study in ClinicalTrials.gov (identifier: NCT03634410), and the protocol is available in open-access [30]. The baseline data were collected from July 2018 until October 2018. For the baseline, a total of 30,000 Danes aged 50 years or older (18,000 employed, 7000 unemployed, 3000 on voluntary early retirement, 2000 on disability pension) were drawn as a probability sample by Statistics Denmark and invited to participate with a personal questionnaire-link via e-Boks (online digital mailbox linked to the Danish social security number). The survey data were merged with high-quality national registers through the unique social security number assigned to all Danish residents at birth or immigration. For the present analyses, only currently employed respondents belonging to International Standard Classification of Occupations (ISCO) groups 1–9 were included. Among those who were employed, the response rate to the entire questionnaire was 56%, but for the present analyses, those replying only partly were included as well, yielding a total sample size of 11,444 employed individuals.

Based on national registers, respondents were stratified into nine occupational groups based on the official Danish version of the ISCO. The International Labour Organization is responsible for the ISCO, which was updated in 2008 [31]. The Danish version of ISCO is a six-digit classification, structured as a five-level hierarchical structure based on information from high-quality national registers at Statistics Denmark, and divides the Danish labor market into 563 professional groups, each containing a number of closely related work functions. The skill requirements in each ISCO group range from I (most basic) to IV (most advanced). For this study, we used the first-level ISCO groups: (1) Managers (levels III and IV skill requirement), (2) Professionals (level IV skill requirement) (e.g., engineers), (3) Technicians and Associate Professionals (level III skill requirement) (e.g., nursing assistants), (4) Clerical Support Workers (level II skill requirement) (e.g., secretaries), (5) Services and Sales Workers (level II skill requirement) (e.g., hair dressers), (6) Skilled Agricultural, Forestry and Fishery Workers (level II skill requirement) (e.g., farmers), (7) Craft and Related Trades Workers (level II skill requirement) (e.g., wood workers), (8) Plant and Machine Operators and Assemblers (level II skill requirement), and (9) Elementary Occupations (level I skill requirement) (e.g., cleaners). Armed Forces Occupations is also an ISCO group (group 0), but was excluded in the present analyses due to a low number of observations. Based on questionnaire replies about the physical work characteristics from each respondent of this study, the majority of ISCO groups 1–4 had seated work (76%, 57%, 74%, and 75%, respectively), and the majority of ISCO groups 5–9 had physical work (86%, 83%, 89%, 72%, and 89%, respectively). For the purpose of the analyses of this study, we therefore grouped ISCO 1–4 together and ISCO 5–9 together.

### 2.2. Measurement of Perceived Attitudes towards Older Workers

The respondents were presented with the question, “Do you think the managers at your workplace think the following about older workers?” and were provided with a list of ten attitudes towards older workers, out of which five were positive and five were negative (see Table 1 for the list of attitudes). The list of attitudes was provided in random order, and the respondents were required to mark the attitudes they believed their managers had (multiple choice).

To further explore the age issue at the workplaces, we included the question, “When are you regarded as ‘old’ at your work workplace?” The respondents were asked to select from a list of ages, starting from age 40, including the option, “There is no age where you are regarded as ‘old’” (see Table 2 for the response options).

### 2.3. Statistics

The SurveyFreq procedure (SAS version 9.4, SAS institute, Cary, NC, USA) was used to produce estimates of prevalence and 95% confidence intervals. The SurveyLogistic procedure (SAS version 9.4) was used to produce odds ratios (ORs) and 95% confidence intervals for chance of choosing each different option of the multiple-choice questionnaires. The analyses were controlled for age, gender, and ISCO group. For gender, men were used as reference, i.e., ORs for women. For ISCO, groups 1–4 were used as reference, i.e., ORs for groups 5–9. Due to the different size and response percentage of subgroups, model-assisted weights were used to produce representative estimates. These weights were used for both the SurveyFreq and SurveyLogistic procedures and were based on information from high-quality national registers at Statistics Denmark [30].

## 3. Results

Across all groups, a larger proportion of the respondents indicated that they believe their managers had more positive than negative attitudes. In general, a relatively low proportion of the respondents indicated that their managers had negative attitudes. The negative attitude that the largest proportion of the respondents believed their managers to have was, “Older workers cannot keep up with the speed and development”. The positive attitude that the largest proportion of the respondents believed their managers to have was, “Older workers’ experience and knowledge is a significant resource for the workplace”. The positive attitudes that the smallest proportion of the respondents believed their managers to have was, “Older workers are productive” and “Older workers are easy to cooperate with” (see Table 1).

When comparing ISCO group 1–4 (mainly seated work) with ISCO group 5–9 (mainly physical work), the results showed that a smaller proportion of the respondents from ISCO group 5–9 pointed at both positive and negative attitudes, and a slightly larger proportion indicated that they did not know. These differences were statistically significant for all the attitudes except, “Older workers are easy to cooperate with”, “Older workers are preoccupied with their retirement”, and “Older workers create conflicts” (see Table 1).

Comparing women and men, there was a general tendency that a smaller proportion of the women reported their managers to have both negative and positive attitudes towards older workers, and a larger proportion of the women reported that they did not know. These differences were significant for all of the attitudes except “Older workers are flexible in regards to working hours”, “Older workers cannot keep up with the speed and development”, and “Older workers should make room for the young” (see Table 1).

Finally, looking at the responses to the question, “When are you regarded as ‘old’ at work workplace?” across all groups the largest proportion of the respondents chose the response option, “There is no age where you are regarded as ‘old.’” Otherwise the most common responses were 60 and 65 followed by 70 years (see Table 2). When comparing ISCO group 1–4 (mainly seated work) with ISCO group 5–9 (mainly physical work), the results showed no notable differences in the proportion of the participants choosing the various response options. When comparing women and men, there was a slight tendency that a larger proportion of women than men reported no age when they are regarded as “old” (see Table 2).

## 4. Discussions

Overall, the results showed that in general a larger proportion of the respondents reported their managers to have more positive than negative perceptions of older workers. Comparing ISCO group 1–4 with 5–9, respondents from ISCO group 5–9 were less likely than respondents from ISCO group 1–4 to report their managers to have both positive and negative perceptions of older workers. Comparing women and men, the women were less likely than the men to report their managers to have both positive and negative perceptions of older workers and more likely to report that they did not know what their managers thought about older workers.

The results showed the general tendency that a larger proportion of the respondents report their managers to have more positive perceptions than negative perceptions of older workers. This indicates that the managers are perceived to be more likely to hold positive than negative attitudes towards older workers, providing some support for previous findings that managers to a large extent do hold positive attitudes towards older workers [15,18,19,20]. Nevertheless, it is generally only about a quarter of the respondents who believe that their managers have the positive perceptions except for the perception, “Older workers’ experience and knowledge is a significant resource for the workplace”, which about half of the respondents point at. Thus, even though the results indicate that only a very small minority of the employees believe their managers to exhibit direct negative attitudes towards older workers, it is still only a minority who believe that their managers exhibit direct positive attitudes as well.

When comparing ISCO group 1–4 (mainly seated work) with ISCO group 5–9 (mainly physical work), employees from ISCO group 5–9 were significantly less likely to report their managers to have four out of the five positive attitudes, the exception being, “Older workers are easy to cooperate with”. These findings support previous studies indicating that the attitudes towards older workers varies across occupational groups [19,21,22,23,24,27] and that managers in white-collar jobs may have less negative attitudes towards older workers [21,22]. Surprisingly, the results showed that respondents from ISCO groups 5–9 were also significantly less likely to believe their managers to have three of the five directly negative perceptions of older workers. The exceptions being “Older workers create conflicts” and “Older workers are preoccupied with their retirement”. Respondents from ISCO groups 5–9 were also more likely to report that they did not know which perceptions their managers had about older workers. There are several possible explanations for these results. It could partly be that the managers’ perceptions of older workers generally are less overt, or the employees are less aware of them in workplaces from ISCO group 5–9. This again may reflect differences in the leadership styles or the relationships between managers and employees. Another explanation may be that the list of both positive and negative perceptions of older workers, presented to the respondents in our study, are more salient to workplaces in ISCO group 1–4, and thus more of the respondents point at these. Either way, the results do indeed indicate that the managers’ perceived attitudes towards older workers differ between ISCO groups 1–4 and 5–9; however, it is less clear from the findings what the differences are. Nevertheless, we do find systematic differences between occupational groups based on a large representative sample, emphasizing the salience of further pursuing this line of research.

When comparing responses from men and women, the results showed the tendency that the women were less likely to indicate that their managers had both the positive and the negative perceptions of older workers. This tendency was significant for four of the five positive perceptions and three of the five negative perceptions. In addition, the women were significantly more likely to respond that they did not know what their managers thought about older workers. These results indicate that there are gender differences between male and female employees’ perceptions of their managers’ attitudes towards older workers. Again, explanations for this pattern of result could include that the women are less aware of or are exposed to the attitudes of their managers or that other attitudes, represented in our study, are less salient for older female workers. However, our data do not allow for conclusions on whether this reflects differences in managers’ attitudes towards older male and older female workers. Further research more explicitly exploring perceptions of and attitudes about older female and male workers, respectively, is encouraged.

Finally, we asked the respondents to indicate at which age they were regarded as “old” in their workplace. The largest proportion of the respondents pointed at the ages of and around retirement. There were no notable differences between the ISCO groups 1–4 and 5–9. We expected that perhaps one would be considered “old” at a younger age in ISCO group 5–9 because of the more physical nature of their work, but our results did not support this expectation. These findings may indicate that it is the retirement age that to a large extent dictate at what age they are considered old at work. When comparing responses from women and men, there was a small tendency for a larger proportion of the women indicating that there is no age when they are considered “old” at their workplace. As mentioned earlier, they were also more likely to indicate that they did not know what their manager thought about older workers. Taken together, these findings could perhaps indicate that age is less of an issue for female workers, and that perhaps other issues are more in focus.

### Strength and Weaknesses of the Study

A major strength of this study is the large, representative sample of workers aged 50 years or older in Denmark. While non-response bias is common in questionnaire studies, all analyses were performed using statistical weights based on high-quality national registers. These weights ensure that the estimates are representative for employees above the age of 50 in Denmark.

Another strength of the study is the internationally accepted way to group occupations into professional groups containing a number of closely related work functions. The Danish version of ISCO is based on objective and high-quality register information from Statistics Denmark and is therefore highly reliable. However, this may also pose a weakness in analyses used in this particular article because the rather rough grouping into the ISCO group 1–4 and ISCO group 5–9 may mask any subgroup variation within these two groups. Nevertheless, we still observed some clear differences in perceived attitudes of managers towards older workers between those with mainly seated work and mainly physical work.

Furthermore, we used an indirect measure of the managers’ attitudes. The advantage of asking the employees about their perceptions of their managers’ attitudes is the avoided risk of socially desirable answers when asking the managers directly. However, these perceived attitudes may not reflect the actual attitudes of the managers. Nevertheless, if older workers perceive their managers to pose negative attitudes towards older workers, whether they are real or not, it is likely to affect their work engagement and motivation to prolong working life; therefore, it is still important and useful knowledge.

Finally, it would have been desirable with a more direct measure of attitudes towards older female and male workers, respectively, and we strongly recommend researchers include this in future studies.

## 5. Conclusions

The results show an overall tendency that a larger proportion of the respondents believed their managers to have more positive than negative perceptions of older workers. Nevertheless, although direct negative attitudes were quite rare, it was still the minority who indicated that their managers had the positive perceptions of older workers. Comparing ISCO groups 1–4 (mainly seated work) and 5–9 (mainly physical work), results showed that employees from ISCO group 5–9 were less likely to indicate that their managers had positive as well as negative perceptions of older workers. These results, based on a large representative sample, indicate that there are indeed differences in the perceived attitudes of the mangers towards older workers between these two ISCO groups, emphasizing the salience of further pursuing this line of research. Finally, the results showed that women were less likely than men to suggest that their managers had positive as well as negative perceptions of older workers, indicating gender differences in perceived attitudes. More research is needed to explore the nature of these differences and, in particular, attitudes towards older female and male workers.

## Figures and Tables

**Table 1:** Proportion of respondents (%) perceiving their managers to hold the respective attitudes towards older workers, divided by ISCO groups and gender as well as odds ratios between these groups.

	ISCO 1–4		ISCO 5–9		OR (95% CI) *	
Do you Think the Managers at Your Workplace Think the Following about Older Workers?	Men (*n* = 3498)	Women (*n* = 3899)	Men (*n* = 2600)	Women (*n* = 1447)	ISCO 5–9 vs. ISCO 1–4	Women vs. Men
Older workers’ experience and knowledge is a significant resource for the workplace	62	56	51	48	**0.68** (0.62–0.74)	**0.85** (0.78–0.93)
Older workers are productive	21	16	15	13	**0.72** (0.63–0.81)	**0.79** (0.70–0.88)
Older workers are flexible in regards to working hours	31	29	23	23	**0.70** (0.63–0.78)	0.95 (0.86–1.05)
Older workers are flexible in regards to work tasks	29	22	25	21	**0.88** (0.79–0.99)	**0.77** (0.69–0.85)
Older workers are easy to cooperate with	22	15	20	14	0.91 (0.81–1.03)	**0.69** (0.61–0.77)
Older workers’ qualifications are outdated	7	6	4	2	**0.46** (0.36–0.58)	**0.77** (0.63–0.92)
Older workers cannot keep up with the speed and development	11	13	10	8	**0.72** (0.62–0.84)	0.98 (0.86–1.13)
Older workers are preoccupied with their retirement	2	2	2	1	0.92 (0.65–1.30)	**0.70** (0.50–0.98)
Older workers should make room for the young	5	4	3	4	**0.74** (0.57–0.96)	0.88 (0.70–1.11)
Older workers create conflicts	2	2	2	1	0.74 (0.53–1.04)	**0.72** (0.53–0.98)
Don′t know	23	29	27	34	**1.22** (1.10–1.36)	**1.34** (1.21–1.48)

* Mutually adjusted for International Standard Classification of Occupations (ISCO) group, gender, and age. Statistically significant ORs are highlighted in bold.

**Table 2:** Proportion of respondents (%) reporting that you are regarded as “old” at the respective ages divided by ISCO group and gender.

	ISCO 1–4		ISCO 5–9	
When Are you Regarded as “Old” at Work Workplace?	Men	Women	Men	Women
40 years	0	0	1	1
45 years	0	1	1	0
50 years	4	4	6	4
55 years	5	4	5	5
60 years	19	14	19	16
65 years	18	17	16	15
70 years	10	12	9	10
75 years	2	2	1	1
80 years or older	1	1	1	2
There is no age where you are regarded as “old”	41	45	40	45

## Data Availability

The authors encourage collaboration and use of the data by other researchers. Data are stored on the server of Statistics Denmark, and researchers interested in using the data for scientific purposes should contact the project leader Prof. Lars L. Andersen, lla@nfa.dk.
